# Phytochemicals in Cancer Prevention and Therapy

**DOI:** 10.1155/2015/324021

**Published:** 2015-12-13

**Authors:** Poyil Pratheeshkumar, Young-Ok Son, Preethi Korangath, Kanjoormana Aryan Manu, Kodappully Sivaraman Siveen

**Affiliations:** ^1^Department of Toxicology and Cancer Biology, College of Medicine, University of Kentucky, Lexington, KY 40536, USA; ^2^Cell Dynamics Research Center and School of Life Sciences, Gwangju Institute of Science and Technology (GIST), Gwangju 61005, Republic of Korea; ^3^Breast Cancer Program, School of Medicine, Johns Hopkins University, Baltimore, MD 21231, USA; ^4^Division of Cancer and Stem Cell Biology, DUKE-NUS Graduate Medical School, Singapore 169857; ^5^Translational Research Institute, Academic Health System, Hamad Medical Corporation, P.O. Box 3050, Doha, Qatar

Despite advances in modern medicine, cancer is still the major cause of mortality in both developing and developed countries. Search for safer and more effective chemoprevention and treatment strategy is a need for the improvement of patient care in the field. Prevention may be more effective and less costly because cancer is largely a preventable disease which could be attributed to a greater extent to lifestyle. Dietary phytochemicals have been used for the treatment of cancer throughout history due to their safety, low toxicity, and general availability. Population based studies suggest that a reduced risk of cancer is associated with high consumption of vegetables and fruits. Promising phytochemicals not only disrupt aberrant signaling pathways leading to cancer but also synergize with chemotherapy and radiotherapy. Thus, the cancer chemoprevention and therapeutic potential of naturally occurring phytochemicals are of great interest. In this special issue we have collected many interesting original research articles and reviews that provide solid evidence to support the application of phytochemicals or dietary agents in prevention and treatment of cancer.

This special issue contains 3 review articles and 9 original peer-reviewed papers. A. M. Harrison et al. performed a systematic review of the biomedical literature for the use of phytochemicals for management of cancer therapy pain in human subjects; X.-Y. Chen et al. explored the molecular mechanisms underlying the antimetastatic activity of black rice anthocyanins and identified its molecular targets in HER2+ breast cancer cells; the study by Y. Zeng et al. reports that Southwest China (especially Yunnan and Tibet) is the center of lowest mortality of cancers in China based on coevolution between human's anticancer activities and functional foods from crop origin center; M. Sugata et al. studied the anti-inflammatory and anticancer activities of Taiwanese purple-fleshed sweet potatoes (*Ipomoea batatas* L. Lam.) extracts; R. Moo-Puc et al. investigated the antiproliferative activity of bonediol, an alkyl catechol isolated from the Mayan medicinal plant* Bonellia macrocarpa* against human prostate tumor cells; B. Moyo and S. Mukanganyama demonstrate the antiproliferative potential of* T. welwitschii* extract on Jurkat T cells; C.-J. Tai et al. report the potential of ethanolic extract of* Taiwanofungus camphoratus* (*Antrodia camphorata*) to enhance the cytotoxicity of cisplatin and doxorubicin on human hepatocellular carcinoma cells; G. Wang et al. explored the molecular mechanism of total flavonoids extracted from* Cotinus coggygria* against glioblastoma cancer in vitro and in vivo; M. N. Mallick et al. studied the anticancer activity of hydroalcoholic extract of* Picrorhiza kurroa* and its fractions; M. F. Abu Bakar et al. demonstrate that the* Garcinia dulcis* fruit extract induced cytotoxicity and apoptosis in HepG2 liver cancer cells; G. Weng et al. reported the curcumin enhanced busulfan-induced apoptosis in leukemia stem-like KG1a cells via downregulating the expression of survivin; S. Kumar and J. Kim in their review discuss potency and selectivity of PLK-1-targeted inhibitors and their molecular interactions with PLK-1 domains.

In conclusion, this special issue discussed the potential anticancer phytochemicals and dietary agents, their molecular targets, and their mechanisms of actions. The understanding of molecular mechanism of a specific plant derived compound against a particular type of cancer will lead to the invention of novel drug and drug targets for therapeutic intervention.

